# Supplementary material for: Abnormal birth weight in urban Nigeria: An examination of related factors
Source: PLoS One. 2020 Nov 24;15(11):e0242796. doi: 10.1371/journal.pone.0242796 (PMC7685448; doi:10.1371/journal.pone.0242796)
Supplement: Attachment — Submitted filename: Reviewer comments ABW in Nigeria.docx [file pone.0242796.s001.docx]

**Abnormal birth weight in urban Nigeria: an examination of related factors**

The authors have used national datasets to explore an important issue of abnormal birthweight – considering not only small babies but also large babies in the context of urbanization.

Methods

- Clarity in your methods that you modeled odds of LBW and HBW separately and not ABW pooled, which is how it reads.

Results

- In the results (i.e., results paragraph 5) you do not need to repeat your methods and just present the results.
- Results on Table 1: remove references to more LBW and more HBW among those who did not receive ANC from skilled providers – your results show there was no significant difference in birth weight based on this variable.
- The results text could be abbreviated to the main points. Paragraphs 1-4 referring to table 1 would be better as one paragraph and summarized to the main highlights.
- Results paragraph 6: The inclusion of individual and health care utilization didn’t decrease the odds of regional variation but the odds ratios were no longer significant when accounting for individual and healthcare variables, except in the North West and North East. Be careful with the language since “X decreases Y” implies a causal relationship. Your description in Paragraph 7 for HBW is much better.
- Results should include the numerical results, you sometimes present them and sometimes do not. Please be succinct and present the main findings and numerical results together (i.e. All included variables for LBW in Model 2 were not significant except lower odds among singleton births (OR 0.19, 95% CI 1.0-0.35, etc.). But also check the results, it appears the 95% CI for LBW Model 2 type of birth has a typo. Paragraph 7 reporting HBW models should also be revised to follow this approach.

Discussion

- The discussion could use some better organization. It was a bit hard to follow the main points you were trying to make. Maybe start the first paragraph of the discussion to highlight your main findings and then unpack each main finding in one paragraph.
- For your first finding… It seemed to me that you are mainly saying: HBW were a bigger concern than LBW in urban Nigeria. You are right in saying we focus much more on LBW in sub-Saharan Africa but with urbanization we need to also be focusing on the issue of overnutrition and the negative implications for newborns. It would be helpful to have some data to support this on the burden of overnutrition in Nigeria to help support this claim for readers who are not so familiar with the data in urban Nigeria.
- Please also make sure each time you refer to studies (i.e., Canada study mentioned in discussion paragraph 1), that you always put the reference.
- There are other factors that can contribute to preterm birth and LBW that are importantly missing from your study (because they are not part of DHS datasets), like maternal infections.
- Shorter birth intervals are often associated with preterm (and thus often LBW) births but your study didn’t find that. Any thoughts why?
- In the paragraph on the region, you talk about the north having lower health statistics in general but many that you are talking about, such as stunting are also directly related to one of your outcomes of LBW. What factors, perhaps not measured in your study, may be accounting for these types of poor health outcomes in the North – instability? Food insecurity?
- You mention in the limitations that you don’t have data on preterm births. That is fine, but Nigeria has a relatively high rate of preterm birth which may be why you are seeing factors we would expect to have an association with LBW – such as maternal undernutrition – not being significant in your study if Nigeria’s low weight births are driven by prematurity rather than intrauterine growth restriction.
- Limitations – should also likely include quality of the birthweight data. In your intro you mention birthweight is often not taken and so you are relying on maternal report of birthweight or review of records when available and so if birthweight is not taken, then mothers may not have had much information to refer to when reporting.

Minor typographical issues to correct. There is no need to respond to these in your response to reviewers, but I am just noting them as small observations I had as I read the paper.

- Consistent use of commas (or not) for reporting values in the thousands in text and tables
- Consistent use of decimal when reporting results (one decimal point or rounded to the whole number)
- When reporting the statistic for significance, please include the “p” rather than just stating the value (i.e., p<0.05);
- Table 1 – the “chi” symbol is not showing up in the table note
- Use “*” to present p<0.05 in Table 1 the same way you do in Table 2 for ease to the reader
- Make sure there is a space between the numbers and the word “Percent”
